# Personalized Therapeutic Strategies in Glioblastoma: Bridging Molecular Markers and In Vivo Observations for Targeted Treatment

**DOI:** 10.3390/biomedicines14102385

**Published:** 2026-10-19

**Authors:** Joaquin Fiallo Arroyo, Jose E. Leon-Rojas

**Affiliations:** 1 Escuela de Medicina, Universidad de Las América, Quito 170125, Ecuador; joaquin.fiallo@udla.edu.ec; 2 Grupo de Investigación Bienestar, Salud y Sociedad, Escuela de Psicología y Educación, Universidad de Las América, Quito 170125, Ecuador

**Keywords:** glioblastoma, personalized medicine, molecular biomarkers, patient-derived xenografts, tumor microenvironment, translational gap

## Abstract

Glioblastoma (GBM) is still the most aggressive primary brain tumor in adults. It has a declining median survival rate and shows strong resistance to treatment. This resistance comes from significant differences within the tumor itself. The past failures of standard treatments have led to a shift in neuro-oncology. We now focus on molecular stratification based on genetic changes that can be targeted, instead of relying solely on histopathological classifications. This review looks at how important molecular drivers, like metabolic changes caused by IDH1/2 mutations, MGMT promoter methylation status, receptor tyrosine kinase signaling involving EGFR and EGFRvIII, and changes in epigenetics seen in H3 K27M, are creating new opportunities in immunotherapy, CAR-T cell therapy, precision radiation, and nanomedicine. We also discuss how preclinical validation through in vivo functional models, including patient-derived xenografts (PDXs) and syngeneic mouse lines, offers key insights into tumor evolution under treatment pressure, how drugs penetrate the blood–brain barrier (BBB), and the immunosuppressive nature of the tumor microenvironment (TME). However, despite the scientific backing for these therapies, recent Phase III clinical trial failures highlight a significant gap in translating these findings into effective treatments. This gap mainly results from random Darwinian clonal replacement and antigen escape. To address this issue, we explore the potential of liquid biopsies, such as cerebrospinal fluid and plasma, for real-time molecular tracking. These can help differentiate between true progression and pseudoprogression. Ultimately, we believe that closing the translational gap requires moving away from static clinical methods and adopting flexible, long-term trial designs. We also need to integrate multi-omic data with artificial intelligence (AI) to predict cellular changes and counteract resistance mechanisms before they lead to clinical recurrence.

## 1. Introduction

Glioblastoma (GBM) remains the most common and aggressive primary brain tumor in adults, characterized by relentless invasiveness and a median overall survival that rarely exceeds 15 months [1]. Despite longstanding efforts in oncology, the clinical reliance on conventional, non-specific therapies has highlighted the limitations of one-size-fits-all therapeutic approaches in the face of a disease characterized by intratumoral heterogeneity and populations of chemotherapy-resistant stem cells [2]. This static approach is essentially ineffective at eradicating the cellular subpopulations that drive the recurrence of the tumor [2]. In response to this impasse, neuro-oncology has undergone a paradigm shift, moving away from the traditional, purely histopathological classification toward a stratification based on molecular signatures and specific genetic alterations [1]. Today, the diagnostic integration of key biomarkers, such as IDH1/2 mutations, MGMT methylation status, the EGFRvIII deletion variant, and H3 K27M mutations, enables the identification of biological phenotypes with exploitable vulnerabilities [1]. In this context, the primary objective of this review is to analyze how these molecular drivers are shaping new directions in targeted therapies, including immunotherapy, CAR-T cell therapy, precision radiation therapy, and nanomedicine. To achieve this, we argue for the need to link the rational design of these therapies with observations derived from in vivo functional models, such as patient-derived xenografts (PDX), syngeneic models, and in vivo imaging techniques [3]. Only through preclinical validation in models that replicate the blood–brain barrier, clonal evolution, and the immunosuppressive microenvironment will it be possible to bridge the significant translational gap that has historically hindered the success of clinical trials.

## 2. Molecular Drivers as Therapeutic Targets

Genomic and epigenomic characterization has transformed neuro-oncology in recent years, teaching us that glioblastoma is not merely a disease but rather a combination of molecular vulnerabilities driven by specific genetic alterations. In this section, we will discuss the main molecular drivers, evaluating their biological rationale, their predictive biomarkers, and their application in clinical practice in the face of tumor adaptive barriers.

### 2.1. Metabolic Dysregulation (IDH1/2)

Mutations in the isocitrate dehydrogenase 1 and 2 (IDH1/2) genes are involved in the pathogenic mechanism of cancer [4]. These IDH1/2 mutations act as initiating oncogenic events by conferring a novel enzymatic activity that converts α-ketoglutarate (α-KG) into the oncometabolite D-2-hydroxyglutarate (2-HG) [5]. The supraphysiological accumulation of 2-HG competitively inhibits multiple α-KG-dependent dioxygenases, notably enzymes of the TET family (DNA hydroxylases) and histone demethylases with a Jumonji domain [5]. This metabolic blockage results in profound epigenetic reprogramming characterized by a CpG island hypermethylation phenotype (G-CIMP), which silences gene expression and halts normal cell differentiation [6]. Studies have cited the genomic detection of the IDH1/2 mutation, often validated via magnetic resonance spectroscopy to detect and quantify levels of the oncometabolite 2-HG in tumor tissue, as a primary predictive biomarker [5,7]. Metabolic inhibition has been clinically validated through the development of compounds such as ivosidenib, enasidenib, and pan-inhibitors with high penetration of the blood–brain barrier, such as vorasidenib (AG-881) [6,8]. Despite the theoretical restoration of cellular differentiation, clinical failure in advanced stages occurs due to tumor evolution; over time, the IDH1 mutation can evolve from an initial driver event into a passenger mutation incapable of sustaining oncogenesis on its own in the face of the acquisition of complex resistance networks [5]. Studies reporting in vivo observations have shown that, in preclinical models of orthotopic xenografts harboring the IDH1 mutation, the administration of targeted inhibitors such as AG-120 achieved effective penetration and strong inhibition of intratumoral 2-HG production, thereby validating the metabolic vulnerability of this phenotype [5].

### 2.2. MGMT Promoter Methylation

The pathogenic mechanism of MGMT is based on the fact that O6-methylguanine-DNA methyltransferase (MGMT) is a key DNA repair enzyme that counteracts the cytotoxicity induced by alkylating agents, such as temozolomide (TMZ), by removing lethal methyl adducts at the O6 position of guanine [9]. Studies have reported its value as a predictive biomarker in the context of glioblastoma, since the standard predictive biomarker for TMZ efficacy is the hypermethylation status of CpG islands in the MGMT gene promoter; an epigenetic process that silences its transcription and reduces the expression of the repair protein [9]. MGMT assessment is critical for stratification, as it has been shown to improve survival when combined with TMZ in elderly patients (eGBM) and those with methylated promoters [10]. However, it has also been reported that clinical inaccuracies in biomarker precision are common due to technical and biological discrepancies between promoter methylation levels (measured via methylation-specific PCR-MSP) and actual MGMT protein expression (assessed via immunohistochemistry-IHC) [9]. Another cause of clinical failure is that resistance to TMZ often arises from secondary defects in the mismatch repair (MMR) system, leading to futile repair cycles that evade apoptosis regardless of MGMT status [9]. Finally, studies conducted using in vivo models of patient-derived glioblastoma xenografts reveal an adaptive evasion mechanism in which continuous treatment with TMZ directly induces upregulation of MGMT expression and enzymatic activity, driving an acquired resistance phenotype [11].

### 2.3. Receptor Tyrosine Kinase Signaling (EGFR/EGFRvIII)

Dysregulation of the kinase pathway, often characterized by amplification of the epidermal growth factor receptor (EGFR) gene, frequently co-occurs with the EGFRvIII deletion mutation, which is constitutively activated independently of ligand binding [12]. The co-occurrence of wild-type EGFR amplification and the vIII mutant transcript constitutes a dual biomarker that defines an intrinsically aggressive tumor microenvironment. Although the kinase is a logical therapeutic target, conventional tyrosine kinase inhibitors and monoclonal antibodies fail in patients with glioblastoma due to limitations in penetration of the blood–brain barrier and robust activation of escape or bypass signaling pathways [12]. The shift toward immunotherapies, such as CAR-T cell therapy targeting the unique EGFRvIII antigen, has also failed to achieve long-term disease control [13]. In vivo observations and initial trials demonstrate that, following infusion of anti-EGFRvIII CAR-T cells, selective pressure rapidly eradicates cells expressing the target, but the tumor undergoes extensive “immune escape”; the cells downregulate the antigen, and the disease recurs driven by subpopulations that retain high expression of wild-type EGFR [12].

### 2.4. Epigenetic Reconfiguration (H3 K27M)

Epigenetic Reconfiguration (H3 K27M) is a mutation exclusive to diffuse midline gliomas (DMG); it is a heterozygous somatic mutation that substitutes lysine for methionine (K27M) in the non-canonical histones H3.3 or H3.1 and acts through a dominant-negative effect [14,15]. The oncohistone H3.3K27M physically sequesters and inhibits the Polycomb Repressor Complex 2 (PRC2), leading to a characteristic global reduction in the repressive trimethylation of histone H3 (H3K27me3) [15]. This epigenetic alteration keeps cells trapped in an oligodendroglial precursor cell (OPC)-like state with a high capacity for self-renewal and blocks normal cell differentiation [16]. The value of this biomarker as a predictive marker stems from the fact that confirmation of the H3 K27M mutation, in conjunction with evidence of global loss of H3K27 trimethylation (H3K27me3), constitutes the diagnostic and predictive standard [14]. In terms of clinical application, ONC201 (dordaviprone) has been shown to penetrate the central nervous system and act as a bidentate antagonist of the dopamine D2/3 receptor and an agonist of the mitochondrial protease ClpP [15]. This molecule is able to metabolically reverse the loss of H3K27me3 and has demonstrated objective clinical activity in recurrent DMG. Despite this progress, long-term treatment failure is driven by intratumoral heterogeneity, as more differentiated astrocyte-like subpopulations can evade the blockade and sustain relapse [15]. In a study using in vivo observations in the immunocompetent RCAS-Tva mouse model, it was reported that direct knockdown using antisense oligonucleotides (ASOs) targeting the H3-3A transcript robustly restored the epigenetic state of H3K27me3; as a result, the glioma ceased its proliferative profile and was forced into physiological terminal differentiation into myelin producing cells (oligodendrocytes), neurons, and mature astrocytes, radically extending the overall survival of the subjects [14]. Table 1 synthesizes the key molecular drivers in GBM.

## 3. In Vivo Observations

### 3.1. Patient-Derived Xenografts (PDX)

To better mirror human disease, treatment response, and prognosis, several studies have used patient-derived xenografts. Their findings are reported in the following section. Regarding the pharmacological response in glioblastoma, PDX models have demonstrated high accuracy in predicting therapeutic response. For example, zebrafish xenografts (zPDX) can rapidly assess in vivo sensitivity to drugs such as marizomib, correlating perfectly with the sensitivity observed in vitro [17]. Furthermore, an innovative “induced recurrence” model in murine PDXs (IR-PDX), which simulates the standard of clinical care (surgery, radiation therapy, and temozolomide), identified patient-specific therapeutic vulnerabilities in relapse; this study detected a gain-of-function mutation in PIK3CA (p.E365K) exclusive to recurrent cells, successfully predicting sensitivity to the inhibitor Alpelisib [2]. Other orthotopic PDX models have validated the efficacy of novel therapies, such as the prolongation of survival through the oncolytic virus SVV-001 combined with radiation. On the other hand, when discussing clonal evolution and heterogeneity under therapeutic pressure, the use of orthotopic PDX models maintained in successive passages (P0–P10) over the years preserves the somatic mutations and transcriptional hierarchy of the original tumor [18]. The same study reports that, under therapeutic pressure from temozolomide (TMZ), overall tumor heterogeneity is reduced, whereas the CT1 population is retained and expands. CT1 denotes a transcriptionally defined, quiescent cancer stem cell population enriched for neural stem cell and quiescent cancer stem cell signatures and underrepresented in proliferative S-phase and mitotic clusters. F3+ does not represent a separate transcriptional cluster; rather, expression of the cell-surface F3 receptor (tissue factor/CD142) is included in the CT1 signature and enables phenotypic enrichment of these cells. Functionally, F3+ cells exhibit substantially greater sphere-forming and self-renewal capacity than F3-negative cells, as well as in vivo tumor-initiating potential. The persistence and expansion of the CT1/F3+ population following TMZ treatment and in recurrent tumors support its role as a treatment-resistant reservoir that contributes to tumor regrowth [18]. Another study also reported that the IR-PDX model revealed that recurrence is phenotypically associated with a transition to a mesenchymal state and with the emergence of a population of cells similar to ciliated neural stem cells [2]. The ability of PDX models to accurately reflect the primary tumor is well established. These mouse models achieve a take rate of 73.9% while preserving histological characteristics and the pattern of diffuse invasion [19]. The correlation in gene expression (RNA-Seq) between the patient’s tumor and the murine PDX ranges from a Pearson correlation coefficient of 0.54 to 0.72 [19]. In predicting tumor-initiating capacity, zPDX models show an almost perfect correlation with mouse PDX models (Spearman’s r = 0.89, *p* < 0.001) [17]. With regard to relapses, the IR-PDX model is capable of capturing more than 60% of the changes in gene expression and more than 30% of the changes in DNA methylation that occur in the patient’s actual relapse [2]. Studies have also reported limitations of these models; conventional murine PDX models take months to develop and have a success rate of only 30–60% [20]. Aggressive tumor infiltration leads to mouse cell contamination ranging from 7% to 68% in the collected tissue, causing genetic “noise” and reducing genomic correlations [19]. The accelerated rate of recurrence in mice (weeks) compared to humans (months) limits the ability to replicate all epigenetic changes (capturing only 30% of methylation events) [2]. Finally, the lack of a true immune microenvironment in the immunodeficient mice used for PDX models limits their usefulness in immunotherapy trials [21].

From a precision oncology perspective, PDX findings could be incorporated into individualized therapeutic planning through a tiered workflow in which tissue obtained during resection undergoes parallel histomolecular characterization and functional drug testing. Candidate therapies could be prioritized when model-derived sensitivity is concordant with the tumor molecular profile and when the agent demonstrates adequate central nervous system penetration and an acceptable clinical safety profile. Recurrence-matched models may be particularly useful for selecting subsequent treatment strategies, as IR-PDX analysis can identify acquired patient-specific vulnerabilities, including the PIK3CA p.E365K mutation and predicted sensitivity to alpelisib [2]. Turnaround time, however, remains a major barrier. Conventional murine PDX establishment requires months and is therefore generally incompatible with treatment decisions during initial postoperative management, although these models may inform treatment at recurrence, subsequent lines of therapy, or eligibility for biomarker-directed trials [20]. In contrast, the reported zebrafish xenograft platform uses a 5-day protocol and can generate treatment-response phenotypes within 72 h [17]. Nevertheless, this assay was performed using established and fluorescently labeled patient-derived cultures, meaning that the experimental duration does not represent the complete interval from surgical resection to a clinical recommendation. Rapid ex vivo single-cell functional profiling of freshly resected tissue could further shorten this interval and may be combined with zPDX screening, while murine PDX models provide longer-term confirmatory validation [20]. At present, these approaches remain investigational and should complement, rather than replace, established histopathological and molecular biomarkers because prospective evidence demonstrating improved outcomes with PDX-guided treatment remains limited [17,20].

### 3.2. Syngeneic Mouse Models and the Tumor Microenvironment (TME)

Studies in mouse models have allowed us to observe cellular interactions in the tumor microenvironment (TME). The TME of glioblastoma exhibits a distinctive spatial segregation: microglia (Tmem119+) tend to adopt an amoeboid morphology and are predominantly located at the invasive periphery of the tumor, whereas infiltrating peripheral monocytes/macrophages (Gal-3+) accumulate within the tumor core [22]. Through the use of mesenchymal stem cells (MSCs) or oncolytic viruses (oAds) that express chemokines (such as CXCL10 or CXCL11), in vivo reprogramming of the TME has been documented, promoting the infiltration of CD8+ T cells, natural killer (NK) cells, IFN-γ-producing cytotoxic T lymphocytes (CTLs), and M1-polarized macrophages, while reducing critical immunosuppressive populations such as regulatory T cells (Tregs), myeloid-derived suppressor cells (MDSCs), and M2 macrophages [23]. Another study showed that there is a sex bias in activation, with male microglia expressing higher levels of major histocompatibility complex class II (MHCII) genes [22]. Research focused on analyzing the mechanisms of immune evasion in glioblastoma has reported that model tumors, such as GL261 or CT-2A, develop multiple evasion pathways. In vivo, the metabolic reprogramming of radiation-resistant glioblastoma cells drives fatty acid oxidation (FAO), which upregulates the expression of the CD47 protein; this mechanism confers direct resistance to macrophage-mediated phagocytosis on tumor cells [24]. Furthermore, monocytes that infiltrate the tumor differentiate into suppressor phenotypes, upregulating the PD-L1 checkpoint and chemokines such as Ccl5 and Ccl22 [22]. Another study reported a counterintuitive finding regarding the TREM2 receptor in myeloid cells: its deficiency in vivo does not slow tumor growth but rather severely impairs the ability of macrophages to phagocytose tumor debris and present antigens via MHC class II, which ultimately drastically reduces the CD4+ T cell-mediated immune response [25]. An advantage of syngeneic models is that, unlike PDX models, syngeneic models (such as GL261, 73C, and CT-2A) in C57BL/6 mice allow for the analysis of the complex interaction between malignant cells and an intact host immune system [21]. This immunocompetence is an essential prerequisite for evaluating combination regimens of radioimmunotherapy, CAR-T cell therapies, immune checkpoint blockade, and oncolytic viruses, as the success of these interventions depends on their ability to remodel a “cold” immunosuppressive microenvironment into a “hot” immunosupportive one mediated by host lymphocytes and macrophages [21,23]. The main limitation of these models is that the dependence of the immune response on specific subpopulations varies dramatically depending on the murine cell line chosen; for example, the GL261 line is rich in neoantigens and relies heavily on CD4+ cells, whereas tumors such as SB28 do not, limiting the generalizability of the findings to all patients [25].

### 3.3. In Vivo Imaging and Blood–Brain Barrier (BBB) Penetration

Two-photon intravital microscopy (2P-IVM), performed through a cranial window in orthotopic models, allows for the real-time, in vivo mapping of the spatiotemporal distribution of vascularization and the microenvironment [26]. This technique has allowed us to track iron oxide nanoparticles labeled with fluorophores following their intravenous administration [26]. Similarly, the 2P-IVM technique made it possible to visualize the cellular dynamics in which myeloid cells (labeled with CX3CR1-GFP) interact closely with and directly phagocytose tumor cell debris (mCherry+) in the living brain over a period of several hours [25]. Other studies have also reported the use of light-sheet microscopy on zebrafish embryos to identify tumor models with direct perivascular invasion [17]. Multiple studies highlight therapies that are able to cross the blood–brain barrier (BBB) in glioblastoma. Firstly, the oncolytic virus SVV-001 effectively penetrates the BBB via intravenous injection due to its selective tropism, successfully infecting central and invasive tumor cells while sparing normal parenchyma [19]. Similarly, it was observed that intravenous administration of anti-CD47 monoclonal antibodies successfully crosses the BBB and binds to glioblastoma cells in in vivo models of post-radiation recurrence [24]. Furthermore, the functionalization of nanoparticles, such as the incorporation of the AP2 peptide, was shown to efficiently exploit transcytosis pathways to increase transendothelial penetration into microvessels toward glioblastoma spheroids, thereby locally concentrating agents such as cisplatin [27]. Furthermore, when we focus on observations of tumor vascular dynamics, in vivo analyses show that, contrary to common belief, residual or early-stage tumor lesions such as those observed in immediate relapses often manage to co-opt and be sustained by normal cerebral blood vessels without immediately disrupting the tight junctions of the BBB [27]. This explains why, at certain stages, peritumoral permeability remains low, thereby protecting the infiltrating tumor [27]. Ultimately, the main limitation of this approach is that two-photon microscopy (2P-IVM) has a severe optical depth limit (approximately 450 μm in the parenchyma) [26]. As a result, it allows only for a superficial volumetric characterization of the tumor, meaning that biological or hemodynamic differences deep within the core of the lesion may go completely unnoticed.

## 4. Novel Directions in Targeted Therapeutics

### 4.1. Immunotherapy and Molecular Signatures

The term “cold” TME in the context of glioblastoma refers to the fact that glioblastoma induces profound immunosuppression characterized by the infiltration of suppressive myeloid cell populations, including macrophages, myeloid-derived suppressor cells (MDSCs), and microglia [3,28]. In addition, there is a systemic reduction and sequestration of naive T cells in the bone marrow, and at the cellular level, T cells undergo severe exhaustion, which exhibits a sex-specific bias, being more pronounced in males and intrinsically mediated by X-chromosome inactivation escape genes such as Kdm6a and UTX [28]. Similarly, following cell therapies, the TME responds with an adaptive increase in the expression of IDO1, PD-L1, TGF-β, and IL-10, along with the recruitment of FoxP3+ regulatory T cells (Tregs) [29]. Clinical trials have also reported failure of PD-1/PD-L1 inhibitors; these studies, which used monoclonal antibodies against PD-1 and CTLA-4, have generally not demonstrated significant improvements in overall survival when used as monotherapy [3]. This is due to the highly immunosuppressive nature of the tumor microenvironment, the tumor’s anatomical immune privilege, and the profound T cell exhaustion phenotype of infiltrating T cells [3]. Some studies discuss strategies for “warming up” the TME, that is, reducing the degree of immunosuppression; these studies propose the use of toosendanin (TSN), a small molecule that reprograms the macrophage immunome by inhibiting the Hck and Lyn kinases [3]. TSN reverses immunosuppression by blocking the differentiation of macrophages into the M2 phenotype, promoting an M1-like proinflammatory phenotype, and reducing levels of IL-10 and TGF-β [3]. This enhances the infiltration and activation of CD8+ T cells and enables therapeutic synergy when combined with immune checkpoint blockade (anti-PD-1 and anti-CTLA-4) [3]. Some limitations that have also been reported in these studies include the fact that the efficacy of immunotherapy is strongly influenced by sex dimorphism; responses differ between males and females due to intrinsic genetic and epigenetic signatures, which complicates the application of universal therapies [28]. Furthermore, the clinical validation of compounds that modulate macrophages (such as TSN) has been conducted in mouse models that do not fully capture the intrinsic heterogeneity of human gliomas; therefore, studies in immunohumanized PDX models are required [3].

### 4.2. CAR-T Cell Therapy

A study focusing on intrathecal bivalent CAR T cells targeting EGFR and IL13Rα2 in recurrent glioblastoma reported that intrathecal infusion of bivalent CAR-T cells simultaneously targeting the EGFR 806 epitope and the IL13Rα2 receptor achieved early reductions in tumor size and decreased MRI enhancement in patients with multifocal disease, although no patient achieved long-term sustained objective responses according to mRANO criteria [30]. In preclinical glioblastoma models, SynNotch CAR-T cells were engineered as a two-step, spatially gated “prime-and-kill” system rather than as constitutively active CAR-T cells. At the molecular level, the SynNotch receptor contains an extracellular single-chain variable fragment that recognizes either EGFRvIII or myelin oligodendrocyte glycoprotein (MOG), a minimal Notch1 regulatory core, and an intracellular Gal4-VP64 transcription factor. Binding of the priming antigen induces proteolytic cleavage of the Notch core, releasing Gal4-VP64 to activate a responsive promoter that drives expression of a tandem CAR. This tandem CAR combines an anti-EphA2 single-chain variable fragment with an IL-13 mutein that preferentially recognizes IL13Rα2 and is coupled to CD8α hinge and 4-1BB/CD3ζ signaling domains. The resulting circuit operates through sequential logic: EGFRvIII, a tumor-specific but heterogeneously expressed antigen, or MOG, a central nervous system-restricted antigen, first licenses the T cell locally. The induced tandem CAR then functions as an OR gate, allowing recognition of cells expressing either EphA2 or IL13Rα2. Because the priming and killing antigens do not need to be expressed by the same cell, activated T cells can eliminate adjacent EGFRvIII-negative tumor cells through trans-killing, thereby reducing antigen escape. EGFRvIII-based circuits restrict activation to tumor regions, whereas MOG-based circuits restrict activation to the central nervous system, where EphA2 and IL13Rα2 distinguish glioblastoma cells from normal brain tissue. Transient and localized CAR expression also reduces tonic signaling and T cell exhaustion while preserving a naive or stem cell memory phenotype. In xenograft models, these circuits achieved complete and durable tumor clearance without detectable off-tumor killing [31]. The molecular design and spatially restricted trans-killing mechanism of SynNotch CAR-T cells are summarized in Figure 1.

CAR-T cells administered intravenously are able to migrate across the blood–brain barrier to active regions of the glioblastoma, reaching higher genomic levels in brain tissue than in peripheral blood [29]. However, they face “antigenic escape”; selective pressure eliminates cells that express the target (such as EGFRvIII), allowing the tumor to relapse driven by EGFRvIII-negative subpopulations [31]. In addition, constitutively expressed CAR-Ts suffer from premature exhaustion due to tonic signaling, an obstacle that SynNotch receptors mitigate by maintaining lymphocytes in a stem cell memory state [31]. In assessing the safety and toxicity profile, a study reported that systemic (IV) infusion of anti-EGFRvIII CAR-T cells did not cause off-target toxicity or severe cytokine release syndrome (CRS) [29]. However, the intrathecal/intraventricular locoregional route of administration (designed to maximize the therapeutic index) caused acute grade 2 and 3 neurotoxicity (immune effector cell-associated neurotoxicity syndrome (ICANS) and tumor-induced neurotoxicity (TIAN)), manifesting as aphasia, muscle weakness, and lethargy [30]. These risks were effectively managed by administering high-dose corticosteroids (dexamethasone) and the IL-1R antagonist anakinra [30]. The main limitation of this approach is that CAR-T cell infusion induces rapid adaptive resistance in the tumor microenvironment (TME), characterized by the overexpression of immunosuppressive molecules (IDO1, PD-L1, IL-10) and the influx of FoxP3+ regulatory T cells, thereby inhibiting long-term efficacy [29].

### 4.3. Precision Radiotherapy and Radiosensitization

Regarding molecular markers and radiosensitization, studies have reported that the inhibition of poly(ADP-ribose) polymerase (PARP) represents a potent mechanistic strategy for increasing the sensitivity of glioblastoma to radiation [32]. In experimental models, molecular inhibitors such as KU-0059436 effectively block the repair of single-strand breaks (SSBs) in DNA induced by ionizing radiation [33]. When the cell enters the S phase (DNA replication), the replication machinery collides with these unrepaired lesions, causing the replication forks to collapse; this generates severe cytotoxic double-strand breaks (DSBs) that make tumor cells entirely dependent on the homologous recombination (HR) repair system for survival [33]. On the other hand, the RNA-binding protein Musashi-1 (MSI1), a regulator of neural stem cells, confers a profile of extreme intrinsic radioresistance by promoting the hyperactivation of the DNA Damage Response (DDR) [34]. Tumor subpopulations enriched for MSI1 exhibit constitutive phosphorylation of the damage transducers ATM and CHK2, as well as overexpression of the RAD51 recombinase [34]. Experimental silencing of MSI1 revokes the tumor’s ability to repair DNA via homologous recombination, exacerbating DNA damage accumulation of γH2AX and inducing high levels of apoptosis when combined with radiation therapy [34]. To enhance therapeutic efficacy while protecting the surrounding healthy parenchyma from irreparable damage, the molecular approach using molecules such as PARP inhibitors is based on a selective principle: the radiosensitization achieved is strictly dependent on cell replication [33]. Because normal brain tissue is non-proliferative, whereas glioblastoma proliferates rapidly, PARP inhibition confers radiosensitization exclusively to tumor tissue without exacerbating neurotoxicity or damaging adjacent neurons [33]. Clinically, this approach has been validated in the Phase 1 PARADIGM trial, which tested the concurrent administration of the inhibitor olaparib alongside hypofractionated radiotherapy regimens (40 Gy in 15 fractions) in elderly patients who were not candidates for curative treatments [32]. The drug demonstrated excellent tolerability and was administered without any measurable adverse effect on the patients’ neurocognitive function (assessed via the Mini-Mental State Examination—MMSE) throughout the follow-up period [32]. Furthermore, it has been reported that fractionating radiation damage using a conventional regimen enhances the synergistic effect with PARP inhibitors compared to using single doses, presumably because prolonged exposure captures a larger proportion of the tumor population as it passes through the replicative phase [33]. The clinical translation of PARP inhibitor therapies administered concurrently with radiation therapy is currently based on early-phase, open-label trials that lack large cohorts and statistical control arms. Furthermore, long-term follow-up to assess cognitive function is limited due to the physical toll of the underlying disease, making it impossible to definitively determine the profile of late-onset neurotoxicity in survivors [32]. The mechanistic effect of blocking PARP could have divergent molecular consequences depending on the cell cycle phase; some data suggest that during the G2 phase, rather than blocking repair, inhibition could indirectly promote interference or defective assembly of alternative repair complexes such as Ku, leading to homologous recombination in a less predictable manner than replication-dependent collapse [33].

### 4.4. Nanomedicine and Targeted Delivery

When discussing smart delivery systems, studies on the subject have reported findings regarding complexes that function as “Trojan horses,” including functional liposomes, polymeric nanoparticles (PLGA, PEG-PLA), dendrimers (PAMAM), inorganic nanoparticles (magnetic iron oxide, silica, gold), and bioengineered protein nanocarriers, notably tHFn(+) human heavy-chain ferritin nanocapsules [35,36,37,38,39]. To cross the BBB and evade its selectivity, nanocarriers are coated with ligands that induce receptor-mediated transcytosis [37]. These systems extensively target the transferrin receptor (TfR), which is highly expressed in both the cerebral endothelium and glioblastoma cells, using transferrin itself, antibody fragments (OX26, RVS10), or synthetic peptides (T7, T12, CRT) [35]. In addition, ligands targeting integrins (αvβ3) via the RGD peptide, folate receptors, interleukin-13 receptors (Pep-1), and CD44 (hyaluronic acid) are used [39]. Some protein nanoparticles, such as tHFn(+), take advantage of the acidic environment of the endosome to disassemble and release genetic material into the cytosol (lysosomal escape) [35]. Studies have shown that modifying the surface with transferrin increases transport across in vitro BBB models by 200% [37]. It is also noted that in vivo, tHFn(+) ferritin nanocarriers loaded with therapeutic siRNA (against TERT and EGFR) cross the BBB, extend the drug’s plasma half-life, and maintain focused concentrations in the brain tumor for up to 24 h, achieving glioma growth inhibition rates of up to 78.9% in orthotopic models [35]. Figure 2 shows a graphical representation of these processes. The limitation of these approaches is that the nonspecific adsorption of plasma proteins following intravenous injection forms a “protein corona” that can mask targeted ligands such as transferrin, thereby inhibiting receptor recognition and severely reducing the efficiency of BBB transcytosis under real-world in vivo conditions [37]. There are also significant regulatory and clinical translation obstacles, as ensuring the reproducibility and industrial scalability of dual formulations and demonstrating long-term chronic toxicity profiles remains complex [39].

Direct comparison indicates that no individual nanocarrier simultaneously provides optimal blood–brain barrier penetration, glioblastoma selectivity, payload versatility, manufacturing reproducibility, and regulatory readiness. Functionalized liposomes and biodegradable polymeric nanoparticles currently have comparatively greater translational feasibility because their composition, pharmacology, and manufacturing processes are better characterized. However, their targeting efficiency depends on ligand density, receptor availability, particle size, and preservation of ligand exposure after formation of the protein corona [36,37,39]. PAMAM dendrimers and inorganic nanoparticles provide high surface functionality and can integrate therapeutic delivery with imaging or magnetic guidance, but concerns regarding cationic toxicity, biodegradation, tissue persistence, and batch reproducibility limit their clinical translation [36,39]. The tHFn(+) ferritin platform demonstrated particularly strong preclinical delivery of siRNA, sustained tumor retention, and up to 78.9% inhibition of orthotopic glioma growth, although it remains at the animal proof-of-concept stage [35]. Transferrin-modified chitosan nanoparticles offer a noninvasive intranasal strategy that circumvents conventional blood–brain barrier transcytosis. Nevertheless, the available evidence primarily demonstrates epithelial permeability and cellular uptake rather than therapeutic efficacy in patients with glioblastoma [38]. Consequently, platform selection should be payload-specific and should consider delivery to infiltrative tumor cells, molecular target engagement, systemic clearance, immunogenicity, scalable manufacturing, and chronic toxicity [36,39]. These comparative characteristics are summarized in Table 2.

## 5. Clinical Translation and Current Challenges

### 5.1. Clonal Evolution

The recurrence of glioblastoma is explained as a Darwinian clonal replacement process, in which therapies create an evolutionary bottleneck that favors the expansion of highly adaptable and resistant subclones [40]. Under the intense therapeutic pressure of the standard protocol, which includes radiation therapy and temozolomide, there is a preferential selection and enrichment of quiescent cancer stem cells identified through transcriptomic profiles such as the CT1 cluster or by the F3 phenotypic receptor, which are intrinsically resistant to treatment and repopulate the tumor after intervention [18]. In addition to simple clonal selection, this therapeutic pressure induces phenotypic and cellular state changes, causing tumors to frequently transition to a mesenchymal state or reprogram their cellular machinery during recurrence [2,40]. The dynamic molecular alterations observed in matched tumors include the emergence of cells resembling ciliated neural stem cells upon recurrence, the loss of original mutations, and the acquisition of compensatory alterations that promote aggressive phenotypes of therapeutic evasion [40].

The main challenge lies in the fact that the evolutionary dynamics of glioblastoma are highly individualized and stochastic, making it difficult to identify resistance mechanisms or mutational alterations that are universal across all patients [40]. Furthermore, relying solely on static approaches based exclusively on DNA or RNA sequencing is insufficient, since many genomic alterations do not translate functionally at the protein level, and the tumor signal is often masked by the infiltration of immune cell populations or stromal cells [40]. At the preclinical and validation stages, the limited availability of truly matched pre- and post-treatment longitudinal tissue samples hinders the systematic and standardized study of this biological progression in a clinical setting [40].

### 5.2. Liquid Biopsies: Real-Time Monitoring via CSF and Plasma

The analysis of liquid biopsies is emerging as a key strategy for monitoring glioblastoma, highlighting significant technical and sensitivity differences between cerebrospinal fluid and blood plasma. Because the blood–brain barrier strongly restricts the release of tumor nucleic acids into the systemic circulation, it has been documented that cerebrospinal fluid, due to its direct contact with the primary tumor, exhibits an enrichment of tumor-derived DNA more than ten times higher than that observed in plasma [41]. These advantages in terms of sensitivity are also reflected in the quantification of circulating tumor cells, whose dynamics can fluctuate in direct correlation with the clinical response or progression of the disease [42]. Because circulating free DNA has a very short half-life, liquid biopsies provide a dynamic, real-time molecular snapshot, allowing for the tracking of genetic or evolutionary changes (“molecular shifts”) throughout the course of treatment [41]. One of the most critical clinical applications of these liquid biopsies is their potential ability to distinguish between true tumor progression and pseudoprogression or pseudo-response; by providing a direct biological and quantitative measure of nucleic acid and cell levels, they help overcome the diagnostic ambiguities arising from inflammatory changes that confound conventional neuroimaging [41].

The primary barrier to clinical translation is the low reproducibility across different studies, a limitation stemming from the lack of standardization in methods for isolating and processing nucleic acids, proteins, and intact cells [41]. Technically, identifying genetic alterations at ultra-low frequencies remains a challenge due to the noise and inefficiencies generated during the initial steps of preparing libraries for enzymatic sequencing [41]. Furthermore, the small sample size of current feasibility studies prevents the definitive establishment of precise quantification thresholds that can be incorporated into clinical decision guidelines to unequivocally distinguish pseudoprogression from tumor recurrence [42].

### 5.3. The Translational Gap in Phase III Clinical Trials

Recent failures in Phase III immunotherapy trials, such as studies involving Rindopepimut or PD-1 blockade therapies (Nivolumab), highlight a significant translational gap caused by mismatches between the methodological design and the complex biology of glioblastoma [43]. Clinical reports explain that drugs fail due to the immense spatial and intratumoral heterogeneity, making it unlikely that therapies targeting a single target will be curative [43]. Intrinsic cellular plasticity and the redundancy of signaling pathways and immune evasion mechanisms enable glioma cells to eliminate or downregulate the target antigen and adapt rapidly under the selective pressure of treatment [43]. Historically, this failure has been exacerbated by the inability of most trials to include agents that successfully cross the blood–brain barrier, the omission of drugs designed to directly target resistant stem cell subpopulations, and the lack of rigorous evidence of “target engagement” in vivo [43]. To address these gaps and bridge the divide, the authors propose moving away from traditional window-of-opportunity trials in favor of structured longitudinal sampling across multiple time points, combined with the use of adaptive platforms that dynamically adjust therapeutic approaches during the study [43].

The design of clinical trials involving longitudinal biopsies faces significant logistical, ethical, and financial challenges, as subjecting patients to multiple stereotactic neurosurgical procedures over time entails significant procedural risks and incurs costs that conventional sponsors typically do not cover [43]. From a methodological perspective, the use of trial designs that allow patients in the placebo group to cross over to the treatment arm (crossover) upon suspected disease progression irreversibly alters the measurements, resulting in a loss of statistical power to assess true overall survival [43]. Finally, the analysis of extremely small tissue samples requires the use of sophisticated “multi-omics” platforms; integrating these platforms introduces a high degree of technical variability, and there are currently no universally standardized statistical tools available to interpret the data without introducing spatial or methodological biases [43]. Collectively, these limitations highlight the need to replace static trial paradigms with a dynamic translational framework integrating adaptive clinical designs, longitudinal molecular monitoring, high-fidelity preclinical validation, and multi-omic analysis, as summarized in Figure 3 [1,43].

## 6. Conclusions and Future Perspectives

The systematic failure of recent Phase III clinical trials has taught us an important lesson, treatment of glioblastoma can no longer rely on single-target therapies when faced with an ecosystem defined by cellular plasticity and its escape pathways. To overcome this persistent translational gap, neuro-oncology must transition to a model in which in vivo functional observations, particularly through longitudinal PDX models and xenografts with real-time monitoring, are established as the gold standard. Only these platforms can capture the complex Darwinian clonal evolution under therapeutic pressure, reliably validating the efficacy of personalized medicine before making the leap to the patient.

However, this preclinical rigor will lack translational impact unless we simultaneously revolutionize our clinical methodology. It is critically important to move away from traditional static approaches in favor of modern, dynamic trial designs. These statistical frameworks provide the clinical agility essential for realistically managing the spatiotemporal heterogeneity of the tumor, allowing interventions to be tailored to the phenotypes that emerge during progression. The ultimate advance for this clinical transformation will be the role of artificial intelligence (AI) and machine learning. By integrating these computational technologies with multi-omic datasets derived from longitudinal fluid and tissue samples, we will be able to decode the intricate immunosuppressive networks of the tumor microenvironment. This convergence will not only refine prognostic models with unprecedented precision but will also grant us the preclinical ability to anticipate cellular reprogramming and adaptive resistance mechanisms long before recurrence manifests clinically. In conclusion, only through the synergy between molecular resolution, the fidelity of translational models, and statistical design will we finally overcome the translational gap and redefine survival in glioblastoma.

## Figures and Tables

**Figure 1:**
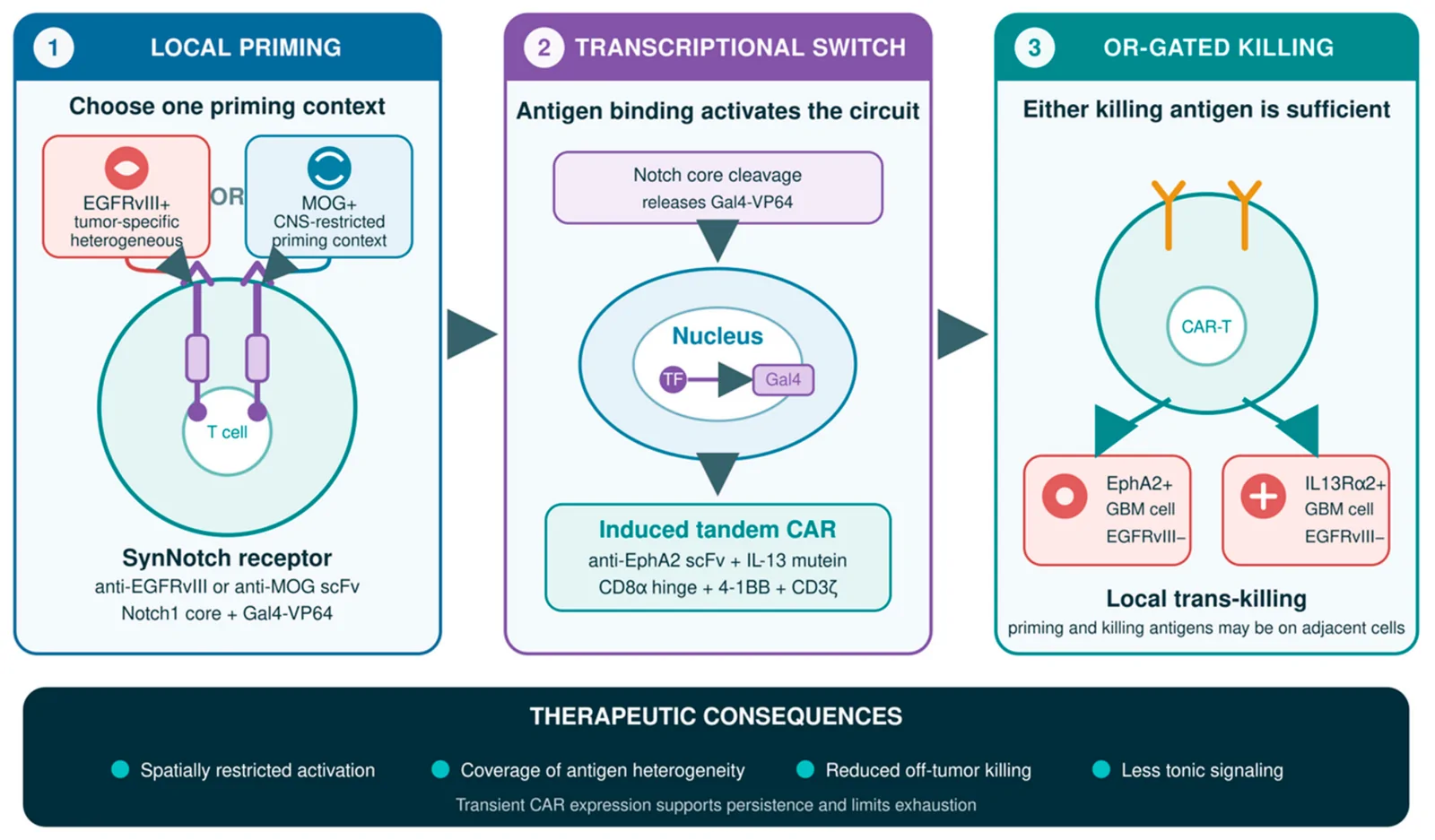
Molecular design and therapeutic logic of SynNotch CAR-T cells in glioblastoma. SynNotch CAR-T cells operate as a spatially restricted, two-step prime-and-kill circuit. Recognition of either the tumor-specific but heterogeneous EGFRvIII antigen or the central nervous system-restricted MOG antigen activates cleavage of the Notch regulatory core and releases the Gal4-VP64 transcription factor. Gal4-VP64 induces expression of a tandem CAR containing an anti-EphA2 single-chain variable fragment and an IL-13 mutein directed toward IL13Rα2, coupled to CD8α, 4-1BB, and CD3ζ signaling domains. The induced CAR functions as an OR gate, allowing elimination of neighboring glioblastoma cells expressing either EphA2 or IL13Rα2, including EGFRvIII-negative cells. This spatially gated trans-killing strategy broadens antigen coverage while reducing off-tumor activity, tonic signaling, and T cell exhaustion [31].

**Figure 2:**
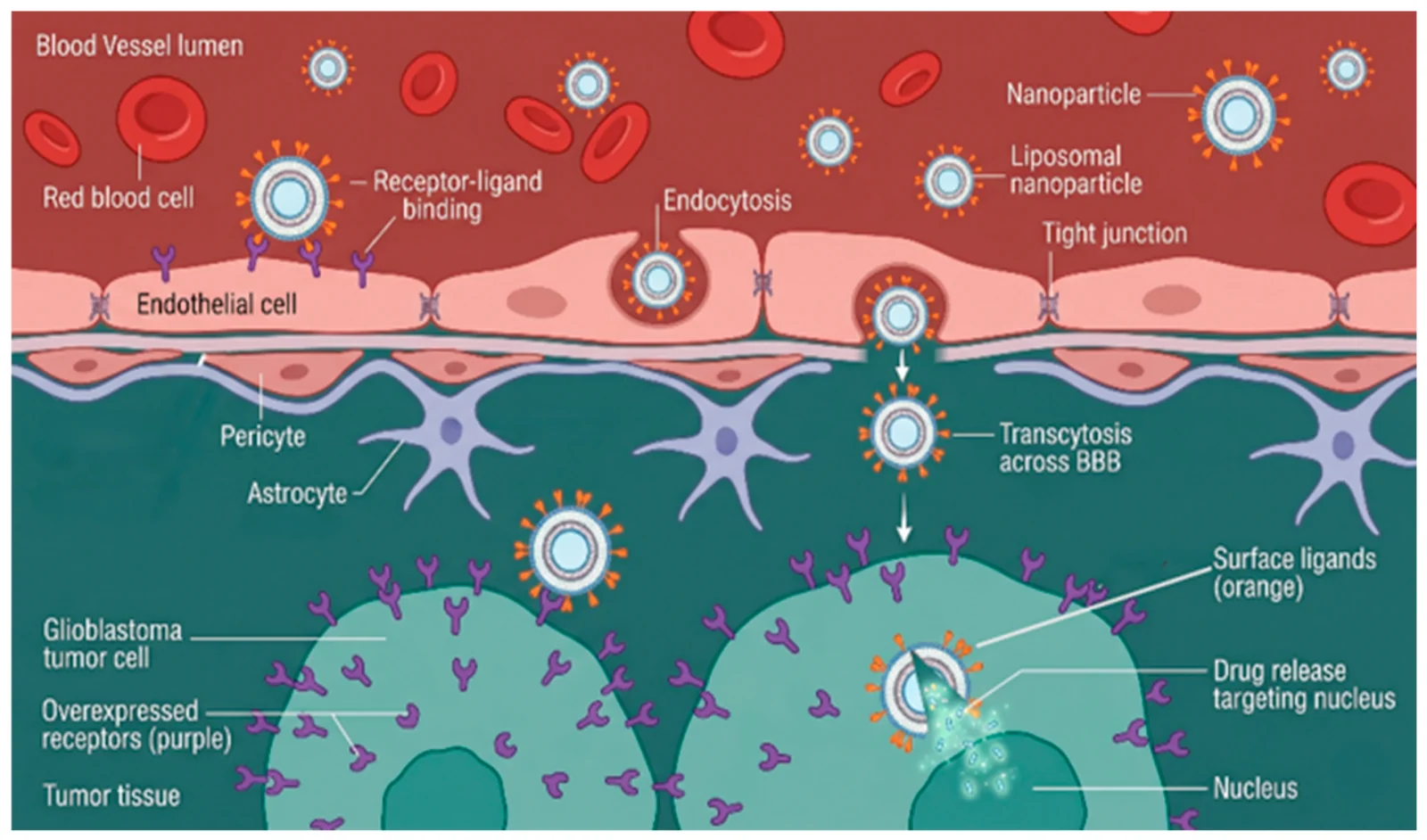
Graphical representation of targeted nanoparticle delivery to glioblastoma cells. The upper panel shows functionalized liposomal nanoparticles navigating the blood vessel lumen. The middle panel illustrates the sequential steps of receptor-ligand binding at the endothelial wall, followed by endocytosis and subsequent transcytosis across the continuous endothelial layer, basement membrane, pericytes, and astrocyte endfeet. The lower panel highlights the selective targeting and intracellular drug release within overexpressing glioblastoma tumor cells.

**Figure 3:**
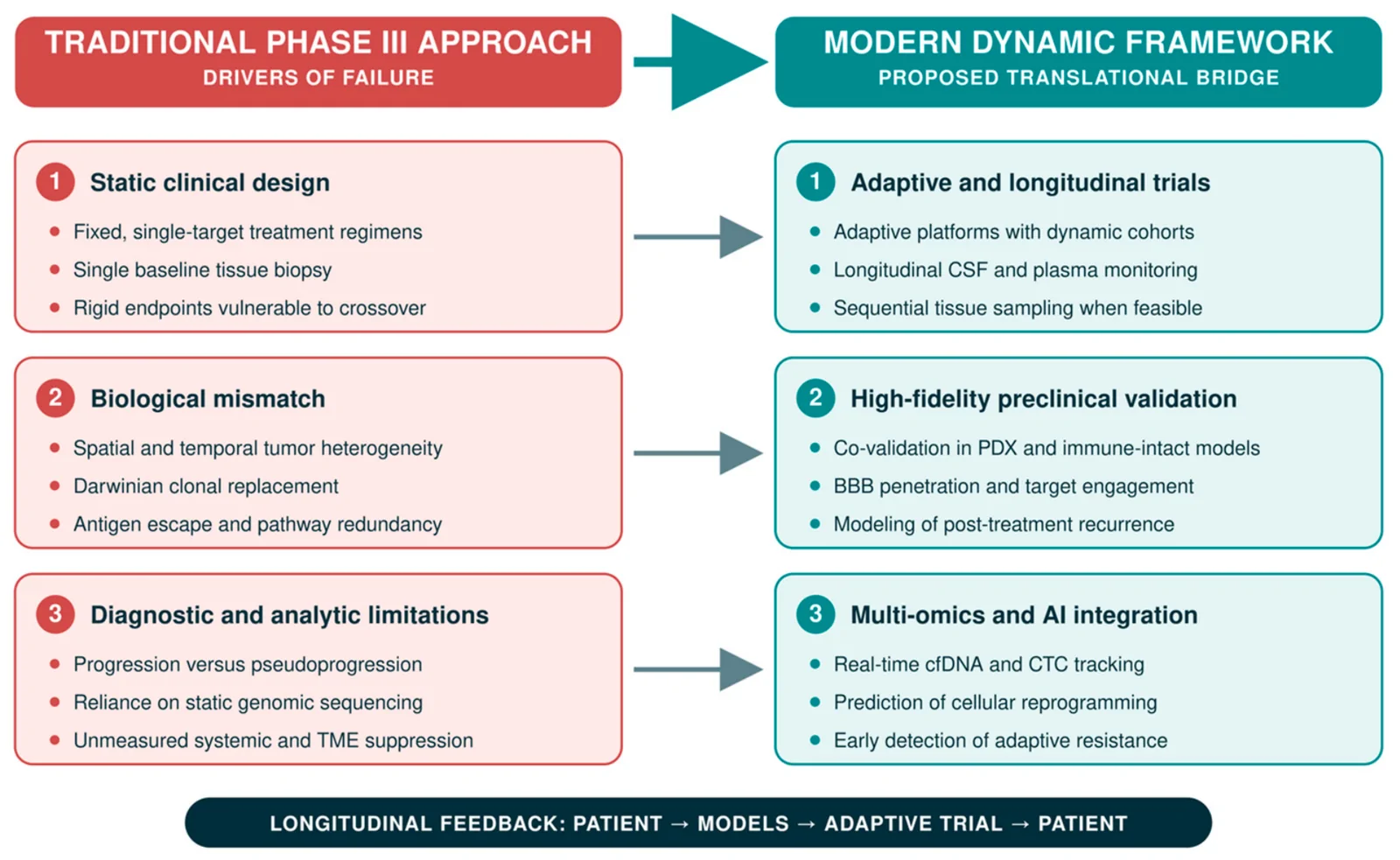
Schematic representation of the translational gap in Phase III glioblastoma trials and the proposed dynamic bridge. Traditional trial designs are limited by fixed single-target regimens, baseline-only tissue characterization, crossover-confounded endpoints, spatial and temporal heterogeneity, antigen escape, uncertain blood–brain barrier penetration, and inadequate distinction between progression and pseudoprogression. The proposed framework integrates adaptive platform trials, longitudinal cerebrospinal fluid and plasma monitoring, sequential tissue sampling, co-validation in patient-derived xenograft and immunocompetent models, confirmation of target engagement, and multi-omic artificial intelligence methods for early detection of adaptive resistance [1,43].

**Table 1:** Principal molecular drivers and clinically relevant biomarkers in glioblastoma and related diffuse gliomas. The table summarizes their underlying molecular alterations, reported prevalence, diagnostic or predictive significance, available or investigational targeted therapies, current clinical status, and major mechanisms of therapeutic resistance.

Molecular Driver	Primary Alteration/Mechanism	Prevalence in GBM	Diagnostic/Predictive Value	Targeted Therapies/Compounds	Current Clinical Status & Resistance Drivers
IDH1/2 Mutations	Somatic point mutations (R132H in IDH1; R172 in IDH2); neomorphic enzymatic activity producing D-2-hydroxyglutarate (2-HG).	~10% (Predominantly secondary GBM/grade 4 astrocytomas)	Favorable prognostic factor; predictive for response to IDH inhibitors and hypermethylation phenotype (G-CIMP).	Vorasidenib (AG-881), Ivosidenib (AG-120), Enasidenib	Vorasidenib demonstrates high BBB penetration; resistance emerges via clonal replacement, metabolic bypass, and loss of driver dependency.
MGMT Promoter Methylation	Epigenetic silencing of CpG islands in the O6-methylguanine-DNA methyltransferase promoter.	~40–50% of primary GBM cases	Primary predictive biomarker for temozolomide (TMZ) sensitivity; strongest prognostic marker in elderly patients.	Alkylating chemotherapy (Temozolomide, CCNU)	Discrepancies exist between promoter methylation (MSP) and protein expression (IHC); resistance driven by secondary mismatch repair (MMR) defects.
EGFR/EGFRvIII	Gene amplification (wild-type EGFR) co-occurring with EGFRvIII in-frame genomic deletion (exons 2–7).	Amplification in ~50%; EGFRvIII variant in ~20–25%	Marker of intrinsically aggressive tumor phenotype and high signaling redundancy.	Tyrosine kinase inhibitors, Anti-EGFRvIII CAR-T cells, Bivalent CAR-T cells	Failure of TKIs due to restricted BBB penetration; CAR-T therapies suffer from rapid antigen escape (loss of vIII) and TME immunosuppression.
H3 K27M Mutation	Heterozygous substitution (K27M) in non-canonical histones H3.3 or H3.1; dominant-negative inhibition of PRC2.	Exclusive to diffuse midline gliomas (DMG); rare in hemispheric GBM	Diagnostic hallmark for DMG; predictive of global H3K27me3 loss and OPC-like stem cell arrest.	ONC201 (Dordaviprone), Antisense Oligonucleotides (ASOs) targeting H3-3A	ONC201 crosses the BBB and acts via dopamine D2/3 and ClpP signaling; long-term failure driven by astrocyte-like subclone differentiation and relapse.

Abbreviations: 2-HG, D-2-hydroxyglutarate; ASOs, antisense oligonucleotides; BBB, blood–brain barrier; CAR-T, chimeric antigen receptor T cell; DMG, diffuse midline glioma; G-CIMP, glioma CpG island methylator phenotype; GBM, glioblastoma; IHC, immunohistochemistry; MMR, mismatch repair; MSP, methylation-specific polymerase chain reaction; OPC, oligodendrocyte precursor cell; PRC2, polycomb repressive complex 2; TKI, tyrosine kinase inhibitor; TMZ, temozolomide.

**Table 2:** Comparative targeting characteristics, translational maturity, and clinical feasibility of nanocarrier platforms proposed for glioblastoma therapy.

Nanocarrier Platform	Targeting and BBB Penetration Strategy	Targeting Efficiency and Supporting Evidence	Translational Maturity and Clinical Feasibility
Functionalized liposomes	Receptor-mediated transcytosis using transferrin or TfR-directed ligands. RGD, folate, IL13R, and CD44 ligands can enhance tumor-cell uptake.	Moderate to high in preclinical models. Transferrin functionalization increased transport across an in vitro BBB model by almost 200%. Efficiency may be reduced by receptor saturation and protein-corona formation [36,37,39].	Comparatively mature carrier class with established pharmaceutical manufacturing experience. GBM-specific targeted formulations remain predominantly preclinical. Ligand stability, off-target uptake, and formulation reproducibility remain barriers.
Biodegradable polymeric nanoparticles, including PLGA and PEG-PLA	PEG prolongs systemic circulation, while TfR, RGD, folate, or other surface ligands facilitate transcytosis and tumor uptake. The polymeric matrix provides controlled payload release.	Moderate and highly tunable. Efficiency depends on particle size, surface charge, PEG density, ligand orientation, and regional BBB heterogeneity [36,39].	Relatively favorable biodegradability and manufacturing feasibility. GBM-specific formulations remain largely preclinical. Challenges include variable drug loading, burst release, and batch-to-batch reproducibility.
PAMAM dendrimers	Small size, multivalent surface functionalization, and ligand-mediated or adsorptive uptake enable delivery of drugs and nucleic acids.	High cellular uptake and payload density in experimental models, but penetration across an intact BBB remains variable and strongly dependent on surface charge [36,39].	Early preclinical development. Clinical feasibility is limited by cationic toxicity, complex synthesis, systemic clearance, and the need for precise surface modification.
Inorganic nanoparticles, including iron oxide, silica, and gold	Magnetic guidance, receptor-targeting ligands, and tumor-penetrating peptides can be combined with imaging, photothermal therapy, or drug delivery.	Strong imaging and theranostic performance in preclinical models, but actual delivery beyond the endothelium and throughout infiltrative tumor regions is inconsistent [36,39].	Primarily preclinical for GBM therapy. Long-term tissue accumulation, limited biodegradability, chronic toxicity, and regulatory complexity restrict clinical feasibility.
Bioengineered tHFn(+) ferritin nanocarriers	Systemic BBB crossing and GBM targeting are combined with pH-responsive disassembly, siRNA release, and lysosomal escape.	High preclinical efficiency. Brain-tumor retention was maintained for up to 24 h, with tumor-growth inhibition of up to 78.9% following delivery of siRNA targeting TERT or EGFR [35].	Early orthotopic animal proof-of-concept. Translation requires reproducible protein manufacturing, immunogenicity assessment, payload standardization, and long-term safety evaluation.
Transferrin-modified chitosan nanoparticles	Intranasal delivery bypasses conventional systemic BBB transport through nose-to-brain pathways. Transferrin facilitates receptor-mediated cellular uptake after passage through the nasal epithelium.	Encouraging epithelial permeability and cellular-uptake results in vitro, but therapeutic efficacy has not been established in GBM animal models or patients [38].	Early preclinical and primarily in vitro. The approach is noninvasive, but nasal clearance, variable deposition, limited dosing capacity, and anatomical differences between experimental models and humans remain important barriers.

## Data Availability

No new data were created or analyzed in this study.
